# Intraoperative radiation therapy (IORT) in soft-tissue sarcoma

**DOI:** 10.1186/s13014-016-0751-2

**Published:** 2017-01-19

**Authors:** Falk Roeder, Robert Krempien

**Affiliations:** 1 Department of Radiation Oncology, University Hospital of Munich (LMU), Marchioninistr. 15, 81377 Munich, Germany; 2 Clinical Cooperation Unit Molecular Radiation Oncology, German Cancer Research Center (DKFZ), Heidelberg, Germany; 3 Department of Radiation Oncology, Helios Clinic Berlin-Buch, Berlin-Buch, Germany

## Abstract

Soft-tissue sarcoma (STS) represent a rare tumor entity, accounting for less than 1% of adult malignancies. The cornerstone of curative intent treatment is surgery with free margins, although the extent of the surgical approach has been subject to change in the last decades. Multimodal approaches usually including radiation therapy have replaced extensive surgical procedures in order to preserve functionality while maintaining adequate local control. However, the possibility to apply adequate radiation doses by external beam radiation therapy (EBRT) can be limited in some situation especially in case of directly adjacent organs at risk with low radiation tolerance. Application of at least a part of the total dose via intraoperative radiation therapy (IORT) with a single fraction during the surgical procedure may overcome those limitations, because radiosensitive structures can be moved out of the radiation field resulting in reduced toxicity while the enhanced biological effectivity of the high single dose improves local control. The current review summarizes rationale, techniques, oncological and functional outcomes including possible pitfalls and associated toxicities based on the published literature for IORT focusing on extremity and retroperitoneal STS. In extremity STS, combination of limb-sparing surgery, IORT and pre- or postoperative EBRT with moderate doses consistently achieved excellent local control rates at least comparable to approaches using EBRT alone but usually including patient cohorts with higher proportions of unfavourable prognostic factors. Further on, IORT containing approaches resulted in very high limb preservation rates and good functional outcome, probably related to the smaller high dose volume. In retroperitoneal STS, the combination of preoperative EBRT, surgery and IORT consistently achieved high local control rates which seem superior to surgery alone or surgery with EBRT at least with regard to local control and in some reports even to overall survival. Further on, preoperative EBRT in combination with IORT seems to be superior to the opposite combination with regard to local control and toxicity. No major differences in wound healing disturbances or postoperative complication rates can be observed with IORT compared to non-IORT containing approaches. Neuropathy of major nerves remains a dose limiting toxicity requiring dose restrictions or exclusion from target volume. Gastrointestinal structures and ureters should be excluded from the IORT area whenever possible and the IORT volume should be restricted to the available minimum. Nevertheless, IORT represents an ideal boosting method if combined with EBRT and properly executed by experiences users which should be further evaluated preferably in prospective randomized trials.

## Background

Soft tissue sarcomas (STS) represent a rare tumor entity, accounting for <1% of adult malignancies [1]. The majority (~60%) are located in the extremities, followed by trunk and retroperitoneal space [2–4]. Surgery with negative margins remains the cornerstone of curative intent treatment, although the extent of the surgical approach has been subject to change in the last decades. Modern treatment concepts in oncology do not only focus on the achievement of local control (LC) and overall survival (OS) but also on preservation of functionality and quality of life [5, 6]. Therefore multimodal organ- and/or function-preserving concepts have increasingly replaced extensive surgical procedures (for example amputations). Within such approaches less extensive surgery with much smaller margins is used resulting in improved functional outcome, but with the need for additional local treatment modalities (usually radiation therapy) to maintain adequate LC. However, in some situations the possibility to achieve LC by additional radiation therapy (RT) can be limited. This is especially true, if adequate doses cannot be applied by external beam radiation (EBRT) alone without considerable risk of severe side effects to surrounding normal tissue, counteracting the aim of the function-preserving overall approach. Application of at least a part of the total dose via intraoperative radiation therapy (IORT) with a single fraction during the surgical procedure might be beneficial in such situations, because radiosensitive structures can be moved out of the radiation field resulting in reduced toxicity while the enhanced biological effectivity of the high single dose improves local control [7–14]. Although STS of different body regions histologically represent the same tumor entities, there are distinct differences in outcome especially between extremity and non-extremity sarcomas [15]. Based on the favourable anatomical situation with less vital structures directly adjacent to the tumor, extremity lesions can usually be resected with much wider margins and surgery results less frequently in residual disease as in other sites, leading to a generally lower rate of local recurrences [15]. Additional radiation is also more limited in non-extremity regions due to nearby structures with low radiation tolerance and salvage surgery in case of local recurrence is also less frequently possible in non-extremity lesions leading to a higher impact of achieving LC in those sites with regard to OS and long-term morbidity [15]. Therefore this review will separately discuss IORT for extremity and non-extremity lesions, focusing on the retroperitoneal space for the latter part. Regarding the literature dealing with IORT in STS, some general aspects have to be kept in mind: For several decades, IORT was available only at a small number of major centers. Therefore randomized or prospective studies on IORT for STS are very rare. Most evidence is based on rather small retrospective analyses with comparably short follow up. Because of the rarity of the disease per se most reports further comprise inhomogeneous cohorts of patients. Therefore comparison of IORT series and non-IORT series is sometimes difficult, although one should be aware that IORT is usually used in patients with rather unfavourable prognostic factors similar to EBRT prior to its implementation as a standard procedure.

### Technique of IORT

Intraoperative radiation therapy is defined as the application of a single fraction of high dose irradiation during surgery. The target volume usually includes the tumor bed after gross total resection or the remaining disease if gross complete resection was not achieved. Usually IORT is used as a boost preceeded or followed by EBRT. Its sole application should be restricted to situations after prior irradiation. An IORT boost offers (at least theoretically) some advantages compared to an EBRT boost: first of all, radiosensitive structures or organs at risk can be effectively spared from radiation exposure by moving them surgically out of the radiation field. The risk for a geographical miss is minimized because target volume definition takes place under visual control. As no substantial intra- or interfractional movements have to be compensated, safety margins can be kept to a minimum and finally overall treatment time is shortened. These advantages have to be weighed against some drawbacks: Usually the final pathological margin will not be available for treatment stratification and the use of a high single dose might result (at least theoretically) in increased late toxicity. Three-dimensional treatment planning is not (yet) available, exact treatment documentation can be challenging and finally doing IORT is still a major interdisciplinary effort and therefore only available at large centers [16].

**Fig. 1:**
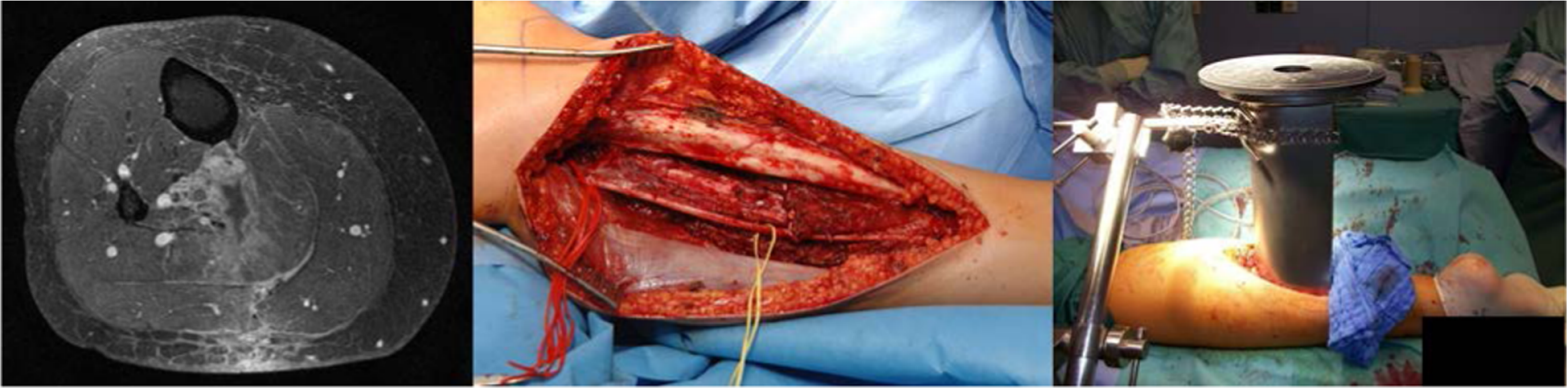
Example of IORT in extremity sarcoma

Technically, two major approaches are in use for IORT treatments of STS: electrons and HDR-brachytherapy. Electrons (IOERT) can be applied either by dedicated conventional LINACs mounted in specialized operation rooms or more recently by small mobile LINACs specifically invented for IORT. After surgical removal of the tumor, the target volume is defined by the radiation oncologist in correspondence with the treating surgeon. Uninvolved radiosensitive tissues can be displaced or covered by lead shielding. An applicator of appropriate size is chosen, manually positioned and attached to the table (see Fig. 1). Applicators are made of steel or plastic to restrict the radiation field laterally and are usually available in different sizes, shapes and bevel angles. Prior to irradiation, the axis of the applicator has to be aligned properly with axis of the LINAC in a defined distance. This can be achieved either by direct linkage between the applicator and the LINAC (so-called hard-docking) or by using a laser-beam guidance system without direct contact between applicator and LINAC (so called air-docking). Most of the LINACs in use are capable of delivering 4–12 MeV electrons (some achieve even 15–20 MeV), thus covering tissue depths of up to 4 cm. The dose is usually prescribed to the 90% isodose. In case of large target volumes, several adjacent applicators might be used. Care should be taken regarding fluids covering the tissue surface or tissue inhomogeneities [16]. Another opportunity is the use of HDR-Brachytherapy. The procedure regarding tumor removal, target volume definition and replacing of radiosensitive organs at risk is very similar to the electron method. Instead of an electron applicator a so-called flab applicator is brought into the operative situs. This usually consists of a flexible (at least to some extent) silicone-based surface mold which includes parallel source guide tubes in a defined distance. The applicator is directly attached to the tissue surface of the target volume, usually fixed with sutures and connected with the HDR remote afterloader. Dwell positions and times are calculated usually based on tabulated values. Dose is generally prescribed at the center of the target to a 0.5 cm depth. Due to the very steep dose fall-off, only tissue depths of 0.5-1 cm can be adequately covered with this technique, however it offer benefits especially if large irregular surfaces have to be covered [16, 17]. Dose concepts are similar between electrons and HDR-brachytherapy. Usually a dose of 10–20 Gy is applied in a single faction. However, the exact conversion of high single doses into biologic equivalent doses in fractionated therapy is still a matter of debate. Using the linear-quadratic equation as the most recognized model, a single dose of 15 Gy would be equivalent to 31–54 Gy in conventionally fractionated RT assuming alpha/beta values of 3–10 for tumor and late reacting tissue response [18, 19]. However the model is not validated for high single doses and its use may result in an overestimation of the equivalent fractionated dose [20, 21]. Further on, the possibility of a direct conversion should be questioned given the growing evidence for a different tissue reaction to high single doses per se if a threshold of 8–10 Gy is exceeded [22]. Based on alternative models [21] and the clinical experience, it seems more reliable to assume an equivalent fractionated dose which is 2–3 fold the IORT dose. Because the tumor effect seems rather 2 fold and the late reacting tissue effect rather 3-fold, organs at risk should be optimally spared and IORT should be combined with EBRT whenever feasible.

### Rationale for IORT in extremity sarcoma

Since Rosenberg et al. [23] showed similar overall survival comparing amputation with limb sparing surgery followed by RT, the combination approach has emerged as the standard of care in extremity sarcomas with high risk features. Subsequent randomized trials [24] and large scale population based analyses [25] have clearly confirmed that postoperative EBRT leads to improved local control in all subgroups. More recently, preoperative EBRT has been proven to be equally effective in terms of LC and OS compared to postoperative EBRT in a randomized trial [26]. However, additional EBRT comes along with increased toxicity. In the postoperative setting, high doses of ≥60 Gy must be applied to large volumes, which can be associated with marked acute and late toxicities and consequently result in unfavourable functional outcomes [27]. In the preoperative setting lower rates of late toxicity have been described [26, 28], which seems to be mainly based on the opportunity to use lower doses and smaller treatment volumes [28]. However, the improvement in late toxicity had to be paid with doubled rates of severe wound complications [26, 28]. The introduction of IORT could offer a smart way out as advocated by several groups [5, 6, 29]. Replacement of the EBRT boost phase by an IORT boost would not only result in smaller treatment volumes because safety margins for daily positioning errors can be omitted, but also in the possibility to exclude organs at risk like major nerves or skin from the radiation field which could at least theoretically reduce late toxicities and improve long term functional outcome. If applied prior to postoperative EBRT with moderate doses, this might avoid an increased wound complication rate and therefore would combine this advantage of the postoperative approach with the smaller treatment volumes known to be beneficial from the preoperative setting [29, 30].

### IORT Series in extremity sarcomas

IORT has been introduced into the treatment of extremity STS in the mid 80s at several US centers [31, 32]. For example, Petersen et al. [32] described the initial experience at Mayo Rochester reporting on 91 patients with limb girdle or extremity STS, which have been treated between 1986 and 1995 with IOERT as a component of therapy. With a median follow-up (f/u) of 3 years, IOERT achieved excellent 3-year LC and OS rates of 92% and 76%, which were at least comparable if not superior to the results achieved with EBRT alone. Disease status (primary vs recurrent) significantly impacted LC (95% vs 81%, p = 0.014) but not OS. Toxicity was prospectively scored according to NCI-IORT criteria with a special focus on neuropathy. Severe neuropathy was observed only in 2% of the patients while 10% developed a moderate form. In the late 80s/early 90s, also some major european centers mainly from Spain (Pamplona, Madrid) and Germany (Heidelberg, Munich) started to use IORT for extremity STS (see Fig. 1). In the following two decades a variety of retrospective single center experiences with only slightly different approaches and similar outcomes have been published (summarized in Table 1) [5, 6, 32–37]. Patient numbers were rather small although most series had mature follow-up (median 33–93 months) [5, 6, 32–36]. Consistently, the cohorts included large proportions of patients with rather unfavourable prognostic factors. For example the rates of R1 resections (usually in the range of 0–25% in non-IORT series) ranged from 17 to 58% [5, 6, 32–37]. Treatment approaches were similar, mainly consisting of 10–20 Gy IORT preceeded or followed by EBRT with 40–50 Gy. With regard to the unfavourable patient cohorts, they consistently reported excellent 5-year-LC rates of 83–90% [5, 6, 35, 36] and 5-year-OS rates of 66–83%, respectively [5, 6, 33, 35, 36]. Moreover, they reported excellent rates (83–100%) of limb preservation [5, 6, 33–36] and good/excellent functional outcome (59–86%) in the vast majority of patients [5, 6, 34, 36]. For example Azinovic et al. [34] treated 45 patients with extremity sarcomas mainly located in the lower limb (82%). 19 (42%) were already in recurrent situation and wide negative margins could be achieved only in 67%. IOERT was administered with a median dose of 15 Gy using mainly 6–9 MeV electrons. 36 patients received postoperative EBRT with 40–50 Gy. With a remarkable median follow-up of 93 months, they reported a crude LC rate of 80%. Surgical margins (5y-LC 87% negative vs 57% positive) and disease situation (5y-LC 88% primary vs 60% recurrent) correlated significantly with LC, while OS was impacted only by disease situation (7-year OS 75% primary vs 47% recurrent). Toxicity was scored according to CTCAE 2.0. Postoperatively delayed wound healing or soft tissue necrosis was found in 18%. Acute radiation toxicity was generally mild and restricted to skin (grade 2–3: 20%). Late toxicity included neuropathy in 5 cases (11%), fracture in 2 (4%), symptomatic fibrosis in 2 (4%) and edema in 2 (4%). The risk for neuropathy was 25% in those with the nerve included into the IOERT field and 11% if not. Median time to neuropathy was 13 months, three of 5 patients showed at least partial recovery after 12 months of duration. Amputation was needed in 5 patients (3 due to toxicity, 2 due to recurrence) resulting in a limb preservation rate of 88%. 31 patients were evaluable for functional outcome of whom 21 (77%) showed no or only minor impairment. Oertel et al. [6] reported another large single center series from the University of Heidelberg. They included 153 patients of whom 25 had resectable distant spread at time of surgery. 92% showed high grade lesions and 50% were larger than 10 cm. 38% were already in recurrent situation and wide negative margins could be achieved only in 49% while 15% even showed gross residual disease. With a median follow-up of 33 months, they reported a 5-year LC and OS rate of 83% in the 128 patients without known distant spread at time of surgery. LC was significantly affected by resection margin and IOERT dose ≥ 15 Gy, while OS was associated with resection margin, grading and IOERT dose. Interestingly, 30% of the local recurrences were found clearly outside the EBRT fields and 40% were judged as marginally while only 10% were located infield-IOERT. They further reported a limb preservation rate of 90% with good functionality defined as impairment not interfering with activities of daily living (ADL) in 86%. Acute toxicity CTCAE 2.0 grade ≥ 2 was observed in 23% (mainly wound healing disturbances) and late toxicity RTOG grade ≥ 2 occurred in 17% including neuropathy in 5%, fibrosis/joint stiffness in 5%, edema in 4% and ulceration in 3%. Callister et al. [37] reported the updated Mayo Arizona experience including 48 patients treated with preoperative EBRT (median dose 50 Gy) followed by surgery and IOERT (median dose 10–15 Gy, 6–9 MeV). Free margins were achieved in 40 patients while microscopically positive margins remained in 8 patients. With a median f/u of 31 months they observed 3-year LC and OS rates of 89% and 75%. Severe postoperative wound complications were found in 16 patients (33%).

**Table 1:** Results of major IORT series in extremity sarcoma

Author	Year	Type	n	f/u	R0	IORT	EBRT	5y-LC	5y-OS	LP	FC
Petersen [32]	1999	r,sc	91	34	n.r.	10–15	45–50	92^b^	76^b^	n.r.	n.r.
Edmonson [33]	2001	r,sc	39	70	62	10–20	45	90^a^	80	95	n.r.
Azinovic [34]	2003	r,sc	45	93	67	15	45–50	80^a^	64^a^	88	77
Kretzler [5]	2004	r,sc	28	52	61	12–15	50	84	66	100	59
Oertel [6]	2006	r,sc	128^c^	33	49	15	45	83	83	90	86
Llacer [35]	2006	r,sc	79	58	42	20 (LDR)	45–50	90	69	100	n.r.
Alvarez [36]	2008	r,sc	53	66	n.r.	7.5–12.5	n.r.	87	75	83	81
Callister [37]	2008	r,sc	48	20	83	10–15	50	83^b^	84^b^	n.r.	n.r.
Roeder [29]	2013	p,sc	34	43	88	10–15	40–50	97	79	94	81
Calvo [40]	2014	r,mc	159	67	84	12.5	45	82	72	94	n.r.
Roeder [30]	2015	r,sc	183	64	68	15	45	86	71	95	83
Roeder [39]	2015	r,mc	259	63	71	12	45	86	78	95	81

*year* year of publication; *type* type of study; *r* retrospective; *p* porspective; *sc* single-center; *mc* multi-center; *n* number of patients; *f*/*u* median follow up in months; *R0* rate of microscopic complete resections in %; *IORT* intraoperative radiation therapy dose in Gy (median or range); *LDR* low dose rate brachytherapy; *EBRT* external beam radiation therapy dose in Gy (median or range); *5y-LC* estimated 5-year-local control rate in %; *5y-OS* estimated 5-year-overall survival rate in %; *LP* limb preservation rate in %; *FC* rate of excellent/good functional outcome in %; ^a^: crude rates, ^b^: estimated 3-year rates, ^c^: excluding patients with distant metastases at time of surgery

Because of the known limitations of the mentioned retrospective analyses, some groups recently focused on different approaches to evaluate IOERT in extremity STS, namely restricted cohorts, prospective evaluations or pooled analyses. Researchers from the University of Heidelberg recently updated their experience but strictly restricted their analysis to patients with extremity (not limb girdle) STS as defined according to WHO, who had received gross complete resection with documented margin and additional EBRT in conventional fractionation with suitable RT documentation available [30]. One hundred eighty-three patients met the inclusion criteria of whom 78% presented in primary situation, mainly located in the lower limb (80%). The majority showed high grade lesions (95%) with advanced stages (IIB-IV:70%). Median IOERT dose was 15 Gy and median EBRT dose 45 Gy. IOERT dose was usually restricted to 10–12 Gy if major nerves had to be included. Median electron energy was 6 MeV. Surgery resulted in free margins in 68% while 32% had microscopically involved margins. With a median follow up of 64 months, the estimated 5- and 10-year LC rates were 86 and 84%. LC was significantly affected by resection margin (5y-LC 92% R0 vs 75% R1) and disease situation (5y-LC 90% primary vs 74% recurrent) in univariate analysis, but only disease situation remained significant in multivariate analysis. The estimated 5- and 10-year OS rates were 77 and 66%. OS was significantly associated with grading, metastases prior/at IOERT and stage in univariate analysis, but only grading and metastases at/prior to IOERT remained statistically significant on multivariate analysis. Toxicity was scored according to CTCAE 3.0. Postoperative complications were documented in 19%, mainly as wound complications. Severe acute radiation side effects were rare (1%), while severe late effects were scored in 20%. This included neuropathy in 8% and fractures in 6%. Secondary amputations were needed in 9 patients, transferring into a limb preservation rate of 95%. Preserved limb function without impairment of activities of daily living was observed in 83%. The authors concluded that IOERT resulted excellent oncological and functional outcome.

The same group recently published also prospective data from a small trial (50 pts) including IOERT as part of local treatment for STS [29, 38]. This single arm study (NeoWTS trial, Clinical Trials.gov NCT01382030, EudraCT 2004-002501-72) evaluated the use of neoadjuvant and adjuvant chemotherapy additionally applied to local treatment in high risk sarcomas. Local treatment included limb-sparing surgery, IOERT and postoperative EBRT. The subgroup of 34 patients with extremity lesions was evaluated separately focusing on local effects [29]. Surgery resulted in free margins in 88% and microscopically positive ones in 12%. Median IOERT dose was 15 Gy and median EBRT dose 46 Gy. With a median f/u of 48 months only one local recurrence was observed, transferring into an estimated 5-year LC rate of 97%. Overall survival was also excellent (5-year rate 79%). Postoperative wound complications occurred in 20%, acute radiation toxicity was generally mild (no grade 3 CTCAE 3.0). Severe late toxicity (CTCAE 3.0) was found in 18%, including only one patient with neuropathy and only one with fracture. Regarding all grades of neuropathy, the rate was 12% in all patients but increased to 25% if only patients with major nerves included into the IOERT area were considered. The final limb preservation rate was 94%. Functional outcome was assessed at different time points in evaluable patients. The cumulative incidence of impairment interfering with ADL including amputation was 83% at one year and 77% at two years.

Another idea to improve evidence for IOERT in extremity sarcoma was to perform pooled analyses of patients from several expert centers. Two groups have performed pooled data analyses so far [39, 40]. The first one, recently published by Calvo et al. [40], included 159 patients from three Spanish expert centers. All presented in primary situation without distant spread. Surgery had resulted in close (<1 cm) or microscopically positive margin but was grossly complete in all patients. IOERT was performed with a median dose of 12.5 Gy and combined with pre- or postoperative EBRT using a median dose of 45 Gy. Median electron energy was 6 MeV. With a median follow-up of 53 months (4–316), the crude local relapse rate was 16%, transferring into estimated 5- and 10-year LC rates of 82% and 81%. 24% of the recurrences were outfield IOERT, resulting in 5- and 10-year central control (infield IOERT) rates of 86% and 85%. Resection margin (R1) was significantly associated with a higher risk for local and infield recurrence. IOERT dose >12.5 Gy was further associated with improved infield IOERT control. Interestingly this effect seemed to be restricted to patients with free margins. Estimated OS at 5 and 10 years was 72 and 64%, significantly associated with age and stage. Severe acute toxicity (RTOG grade ≥ 3) was described in 14% mainly as skin reactions and wound healing disturbances. Severe late side effects (RTOG grade ≥ 3) were reported in 10%, mainly neuropathy.

The second pooled analysis, which has been recently published in abstract form [39], included patients from three european centers (Heidelberg, Madrid, Aviano). After a first attempt including 320 patients, which had been presented at the ISIORT meeting 2008 in Madrid [41], the authors decided to tighten the inclusion criteria similarly to the above mentioned latest series from Heidelberg because of large inhomogeneities in the cohort. The actual analysis, presented at the ISIORT meeting 2015 in Barcelona [39], comprises 259 patients with extremity STS (as defined by WHO criteria) who received at least gross complete resection, IOERT and additional EBRT. The cohort includes 20% patients already in recurrent situation and 29% patients with microscopically positive margins. Median IOERT dose was 12 Gy and median EBRT dose 45 Gy. With a median follow up of 63 months, the crude local failure rate was 10%, transferring into an estimated 5-year LC rate of 86%. Resection margin (5-year LC 94% R0 vs 70% R1) and disease situation were significantly associated with LC in univariate analysis, but only resection margin remained significant on multivariate analysis. Estimated 5-year OS was 78%, which was significantly influenced only by grade and stage IV prior or at IOERT. Secondary amputations were needed in 5%, mainly due to recurrence. Functional outcome was rated as good (not interfering with ADL) in 81% including and 86% excluding amputations.

In summary, the combination of limb-sparing surgery, IORT and EBRT resulted consistently in excellent 5-year LC rates of 82–97% [5, 6, 29, 30, 35, 36, 39, 40] in patients with extremity STS. Those results are at least equal to major Non-IORT series, which consistently report 5-year LC rates of 83–93% [15, 42–50], especially if the higher proportions of patients with unfavourable prognostic factors in the IORT series are taken into account. Beside from oncological outcome, IORT containing approaches resulted consistently in very high limb preservation rates (83–100%) [5, 6, 29, 30, 33–36, 39, 40] with good functional outcome (59–86%) [5, 6, 29, 30, 34, 36, 39]. This might be attributed to the smaller high dose volume compared to an EBRT boost as treatment volume was clearly associated with increased late toxicity in a randomized trial using EBRT alone [28]. Some questions regarding supposed and actual IOERT-associated toxicities should be additionally addressed: Postoperative complications, especially wound complication rates are similar in IORT- and Non-IORT containing approaches. In the largest single center series from Heidelberg, postoperative complications (CTCAE 2.0) were found in 18% of the patients using mainly postoperative EBRT [30]. In the prospective trial, postoperative complications of all grades (CTCAE 3.0) were found in 20% of whom only 9% were grade 3 using a similar approach [29]. Calvo et al. [40] observed an even lower rate of 5% wound complications in their pooled analysis and Kunos et al. [51] found a 15% rate if IOERT was combined with postoperative RT and 36% if IOERT was combined with preoperative IOERT. Those figures almost exactly equal the numbers from the NCIC trial comparing preoperative and postoperative EBRT without IORT, which reported 35% in the preoperative and 17% in the postoperative arm [26] using almost identical definitions of wound complications. Thus, it seems unlikely that IOERT increases the wound complication rate per se. Second, neuropathy has been considered as a dose limiting late toxicity for IORT containing approaches based on the experience from other body regions [52]. However Roeder et al. [29] observed 12% neuropathy of all grades (CTCAE 3.0) including only 3% grade 3 in their prospective subgroup analysis. Azinovic et al. [34] found 11% neuropathy in total in their series and Calvo et al. [40] reported a 3% RTOG grade 3 neuropathy rate in their pooled analysis. These neuropathy rates seem lower than historical reports from other body regions which might be attributed to the fact that most of the expert centers try to exclude major nerves from the IORT area in extremity sarcoma whenever possible. If only the patients are considered in whom major nerves have been included into the IORT fields, the rates of neuropathy (all grades) increased to 25% in both series by Roeder et al. [29] and Azinovic et al. [34] including a nearly three-fold increase in grade 3 neuropathies. Although not shown in the mentioned series, the dose-dependency of neuropathy has been established for IORT long ago in other body sites. Gundersson et al. [53] described 3% NCI-IORT grade 2/3 neuropathy with IORT doses ≤12.5 Gy compared to 21% with ≥ 15 Gy in a series of patient with colorectal cancer. Haddock et al. [54] recently confirmed a significant increase in neuropathy if a treshold dose of 12.5 Gy is exceeded. Therefore major nerves should be excluded from the IORT field whenever feasible or the dose should be limited ≤12.5 Gy. On the other hand one has to keep in mind that the alternative with regard to treatment radicality would be to sacrifice the corresponding nerve surgically in most of the mentioned situations, which would results in a severe neuropathy rate of 100%. Finally, IORT might result in increased fibrosis. Van Kampen et al. [55] thoroughly analysed the association between fibrosis (scored according to LENT-SOMA criteria) and IOERT combined with EBRT in 53 patients. They found a 21% rate of fibrosis of all grades and a 9% rate of severe fibrosis. In a subsequent Cox model only the IOERT volume was significantly associated with severe fibrosis. While an IOERT volume of 200 ccm was associated with a 5% risk of severe fibrosis, the risk increased to almost 50% if the volume was doubled. Thus, the IOERT volume should always be limited to the possible minimum. However, IOERT as part of a multimodal approach offers excellent outcomes in patient with extremity STS even in prognostic unfavourable situations. IOERT is associated with low acute and late toxicity and results in high limb preservation rates with good functional outcome if the mentioned issues are properly considered and seem therefore beneficial compared to EBRT alone at least in subgroups.

### Rationale for IORT in retroperitoneal sarcoma

Similarly to extremity STS, surgery remains the cornerstone of curative intent treatment in retroperitoneal sarcoma [56, 57]. However, in contrast to extremity sarcoma, local progression remains the dominant pattern of failure with roughly 50–80% of the patients failing locally even after gross total resection [52, 58–62]. Resection margin is a strong prognostic factor [59–61] but wide margins are usually not achievable [19, 56, 57, 60]. This builds up (at least theoretically) an even stronger rationale for the addition of radiation therapy than in extremity sarcoma. But although retrospective comparisons consistently show improved LC rates with the addition of radiation [59, 60, 62], a clear survival benefit has not been proven and a randomized comparison of combined modality treatment vs surgery alone is still missing. Further on, postoperative irradiation of the tumor bed is often limited by the tolerance of surrounding organs at risk [52]. Based on the experience in extremity STS and retrospective data, doses of 60–70 Gy would be needed in the postoperative setting to achieve adequate LC especially regarding the narrow surgical margins [19, 63, 64]. However, tumor cavities after resection of retroperitoneal STS are usually large and subject to considerable inter- and intrafractional movement. Applying such doses with the generous safety margins known from extremity sarcoma would result in excessive toxicity as the tolerance dose for small bowel is only about 50–55 Gy in small volumes [52]. These limitations have led to an early interest in the use of IORT in addition to postoperative radiation already in the late 80s. The NCI conducted a small randomized trial which compared the combination of an IOERT boost (20 Gy) with moderately dosed postoperative EBRT (35–40) Gy versus postoperative EBRT alone using 50–55 Gy [52]. After inclusion of 35 patients and a medium follow up of 8 years, they observed a significantly improved LC rate of 60% vs 20% in favour of the IOERT arm. Late gastrointestinal toxicity (scored according to NCI-IORT criteria) was also significantly lower (13% vs 60%) but the neuropathy rate was clearly increased (60% vs 5%) with the use of IOERT. Several other groups have reported also encouraging LC rates with the combination of IORT and postoperative EBRT in retrospective single-center analyses [19, 65, 66]. For example Alektiar et al. [66] reported on 32 patients, of whom 23 had been enrolled in a phase I/II trial evaluating the combination of 12–15 Gy HDR-IORT followed by EBRT with 45–50.4 Gy and 9 patients had been treated accordingly but off protocol. About two thirds of the patients suffered already from recurrent disease, the majority had high grade tumors and the most common histology was liposarcoma. Gross total resection was achieved in 30 patients, while the remaining two had minimal gross residual disease. They observed an encouraging 5-year local control rate of 62% and a 5-year overall survival of 45%. The overall complication rate (scored according to NCI-IORT criteria) was 34%, mainly represented by gastrointestinal obstruction (18%) and fistula formation (9%), while the rate of neuropathy was only 6%.

However, with a closer look to the reported results, central (infield IORT) local control was usually much higher than overall local control. For example Krempien et al. [19] analysed 67 patients who had been treated with IORT with or without additional postoperative EBRT at the University of Heidelberg. Rates of chronic gastrointestinal toxicity (10%), neuropathy (8%) and ureteral stenosis (3%) scored according to RTOG criteria were considerably low, and although most patients showed microscopically incomplete resection (51%) and 18% even suffered from gross residual disease, they observed a 5-year central control rate (infield IORT) of 72%. However, regarding local control (defined as re-growth or progression inside the abdominal cavity) the 5-year rate dropped to only 40%, indicating that many local failures did not occur in the high risk region covered by IORT but in the adjacent low risk region. Thus, the combination of IORT and EBRT seemed effective in sterilizing the high risk region in most of the patients, but postoperative EBRT alone seemed not able to control residual disease in the adjacent low risk regions probably due to the known limitations in dose and target volume coverage. This raised the question if preoperative radiation with or without IORT might be beneficial.

Compared to the postoperative approach, preoperative radiation therapy can offer several benefits, including a more precise target volume definition with smaller safety margins, reduced dose to adjacent organs at risk because of their displacement through the tumor itself, a possible devitalisation of tumor cells prior to surgery, fibrosis and thickening of the pseudocapsule, at least moderate tumor shrinkage and the avoidance of treatment delays due to postoperative complications [56, 58, 67]. This should result at least theoretically in less toxicity due to reduced doses in adjacent organs at risk but increased local control due to a more adequate target coverage which could be further enhanced by an intraoperative boost.

Several groups have evaluated combinations of preoperative and intraoperative radiation therapy and consistently reported high local control rates with acceptable toxicities (see Table 2) [57, 67–73]. For example Petersen et al. [57] reported the Mayo experience with 87 patients, who have been treated with preoperative EBRT (mainly 45–50 Gy) followed by maximal resection and IOERT (median dose 15 Gy). About half of the patients presented already in recurrent situation, mainly with large (median size 10 cm) high grade tumors (62%). Most patients had at least microscopically incomplete resections (64%) while 17% showed even gross residual disease. Nevertheless, they observed an encouraging 5-year LC rate of 59% and a 5-year OS rate of 48%. Resection margin had a strong impact on local control and overall survival. Severe gastrointestinal toxicity (scored according to modified NCI-IORT criteria) was found in only 18% and severe neuropathy in only 10% of the patients. To further evaluate the benefit of IORT after preoperative EBRT and surgery several retrospective comparisons have been performed but resulted in inconsistent findings. Gieschen et al. [68] reported on 29 patients from MGH, who had received preoperative EBRT (median 45 Gy) and gross complete resection and were treated either with 10–20 Gy IORT or no further therapy. They observed a clearly improved 5-year LC rate of 83% with IORT compared to 61% in patients without IORT. Moreover they described a significantly different 5-year OS rate of 74% vs 30% favouring patients with additional IORT treatment. An update of the MGH experience published by Pierie et al. [69], which included 62 patients receiving preoperative EBRT followed by surgery with/or without IORT confirmed the results of the initial analysis. The group who received additional IORT showed a 5-year OS of 77% compared to 45% in patients without IORT. According to multivariate analysis, IORT was an independent prognostic factor regarding both local control and overall survival. In contrast, Ballo et al. [71] did not observe a significant benefit for the addition of IORT in their analysis of 82 patients who had received pre- (60%) or postoperative EBRT (40%) and gross compete resection. They reported a 5-year LC rate of 51% in the IORT group compared to 46% the non-IORT group. According to multivariate analysis, resection margin and primary vs recurrent situation were identified as strong prognostic factors for local control. However, those factors were clearly overrepresented in the (much smaller) IORT group (R1: 61% vs 43%, recurrent situation 40% vs 25%), and therefore a bias cannot be fully ruled out.

**Table 2:** Results of major series IORT series in retroperitoneal sarcoma

							EBRT		IORT			
Author	Year	Type	n	f/u	GTR	pre	post	dose	%	dose	5y-LC	5y-OS
Sindelar [62]	1993	p,sc	15	96	100	-	100	35-40	100	20	60^1^	45^2^
		ran	20		100	-	100	50-55	-	-	20^1^	52^2^
Alektiar, [66]	2000	r,sc	32	33	94	-	78	45-50	100	12-15	62	45
Gieschen [68]	2001	r,sc	16	38	100	100	-	45	100	10-20	83	74
			13		100	100	-	45	-	-	61	30
Petersen [57]	2002	r,sc	87	42	83	75^b^	28^b^	48	100	15	59	48
Bobin [76]	2003	r,sc	24	53	92	29	63	45-50	100	15	46^1^	56
Krempien [19]	2006	r,sc	67	30	82	-	67	45	100	15	40^3^	64
Pierie [69]	2006	r,sc	14	27	100	100		40-50	100	10-20	n.r.	77
			27		100	100		40-50	-	-	n.r.	45
Pawlik [70]	2006	p,mc	72	40	75	100	-	45	47	15	60^4^	50
Ballo [71]	2007	r,sc	18	47	100	60	40	45-66	100	15	51	n.r
			63	47	100			45-66	-	-	46	n.r
Sweeting [72]	2013	r,sc	18	43	100	94	-	45	100	10-20	64	72
Gronchi [73]	2014	r,mc	83	58	84	88	-	50	17	12	63^1,5^	59
Roeder [67]	2014	p,sc	27	33	96	100	-	45-55	85	12	72	74

*year* year of publication; *type* study type; *p* prospective; *r* retrospective; *ran* randomised; *sc* single center; *mc* multicentre; *n* number of patients; *f/u* median follow-up in months; *GTR* percentage of patients in whom gross total resection was achieved; *pre* percentage of patients with preoperative EBRT; *post* percentage of patients with postoperative EBRT; *dose* EBRT dose in Gy (median or range); *IORT* intraoperative radiation therapy; *%* percentage of patients who received; *IORT* dose: dose of IORT in Gy (media or range); *5y-LC* estimated 5-year local control in rate if not otherwise specified; *5y-OS* estimated 5-year overall survival if not otherwise specified; *n.r.* not reported; ^1^: crude rate; ^2^: median OS in months; ^3^: abdominal control; ^4^: in grossly resected patients; ^b^: 14% received both (pre- and postop. EBRT with lower doses (included in pre- and postop figures); 11% received no EBRT; ^5^: in resected patients

**Fig. 2:**
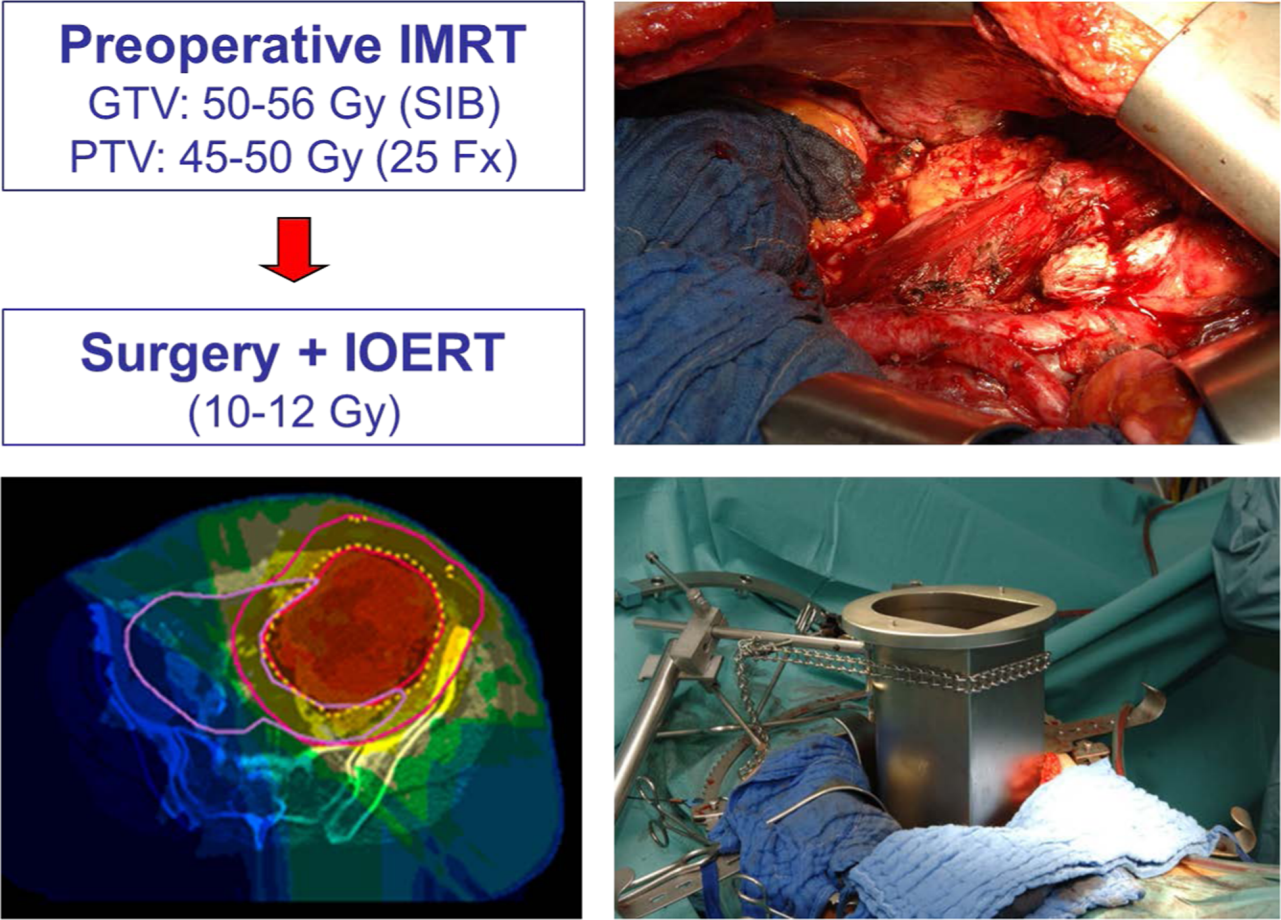
Treatment of retroperitoneal sarcoma according to RETRO-WTS trial [56, 67]

The combination approach is further currently evaluated in a prospective single arm trial (Retro-WTS trial, Clinical trial number NCT01566123, see Fig. 2) at the University of Heidelberg [56, 67]. Patients are eligible if they suffer from retroperitoneal sarcoma of any grade with a size ≥5 cm, are free of distant metastasis and deemed at least marginally resectable. Treatment consists of preoperative intensity-modulated image-guided radiation therapy using the simultaneously integrated boost technique up to 50–56 Gy followed by surgery and intraoperative radiation with 10–12 Gy. The primary endpoint is 5-year local control. Secondary endpoints include progression-free survival, overall survival and toxicity. Due to slow accrual, an unplanned interim analysis was recently performed after 27 patients with a median f/u of 33 months [67]. Patients showed typical features of retroperitoneal sarcomas with a median size of 15 cm, mainly high grade lesions (82%), predominantly liposarcomas (70%) and 15% already in recurrent situation. Neoadjuvant IMRT was completed as planned in 93%. Surgery was gross complete in all except one patient but resulted in microscopically positive margins in 74%, although contiguous organ resection was used in 96%. IORT was performed as planned in 85% with a median dose of 12 Gy using a median energy of 8 MeV. Local failures (defined as intraabdominal recurrence) were observed in 7 patients (crude rate 26%), resulting in an estimated 5-year local control rate of 72%. Recurrent situation was the only significant negative prognostic factor (estimated 5-year local control 88% in primary situation). Distant failure was the main reason for progression (5-year DC 63%) with histology of leiomyosarcoma being the only significant negative prognostic factor. Estimated 5-year overall survival was 72%. Acute radiation related toxicity was quite acceptable (CTCAE 3.0 grade 3: 15%), mainly haematological or gastrointestinal. Postoperative complications were considerable (Clavien-Dindo grade ≥ 3: 33%) with a relaparotomy rate of 15% but mainly related to surgery. 30 day mortality was 0% but two patients died in the prolonged postoperative period. Severe late toxicity (CTCAE 3.0 grade 3) was very rare with 6% at 1 year and 0% at 2 years in evaluable patients.

In summary, the combination of preoperative EBRT, surgery and IORT resulted consistently in high 5-year local control rates of 51–83% in patients with retroperitoneal STS (see Table 2) [57, 67, 68, 70–73]. Those results seem to be superior to surgery alone or surgery combined with EBRT at least with regard to local control [52, 67, 74] and in some reports even to overall survival [67, 68]. Further on, preoperative EBRT in combination with IORT seems to be superior to the opposite combination with regard to local control and toxicity [67, 71, 74]. Some groups reported excellent central (infield IORT) local control rates with acceptable toxicities using IORT and postoperative EBRT [19, 52], but locoregional (abdominal) control was rather poor [19], although the toxicity profile was superior to postoperative EBRT alone [52]. This might reflect the general limitations of the postoperative approach in target coverage/dose intensity and/or the difficulties in adequately sparing adjacent organs at risk (especially small bowel). In the randomized NCI trial, severe chronic GI-toxicity (scored according to NCI-IORT criteria) was found in 13% in the IORT + limited EBRT arm versus 50% in the postoperative EBRT only arm [52]. Krempien et al. [19] similarly described bowel stenosis/fistula in 11% using IORT and postoperative EBRT with moderate doses. Petersen et al. [57] observed severe GI-toxicities (modified NCI-IORT criteria) in 12% combining preoperative EBRT, surgery and IORT and Roeder et al. [67] observed severe late toxicities (CTCAE 3.0) only in 6% (although with short follow-up) in their prospective trial using preoperative IMRT, surgery and IORT. Nevertheless, gastrointestinal structures should be excluded from IORT fields whenever possible and adequately spared during preoperative radiation therapy using modern EBRT techniques. In the earlier mentioned NCI trial, neuropathy was the major toxicity in the IORT arm. While only 5% of the patient in the postoperative RT only arm had neuropathy, it was found in 60% of the IORT arm [52]. As known from IORT studies dealing with colorectal cancer, high single doses (>15 Gy) during IORT can be associated with a considerable risk for neuropathy [53, 54]. In the NCI trial most patients received a single dose of 20 Gy [52]. Further on, many patients had probably been treated with overlapping fields which would results in even higher doses probably responsible for the high neuropathy rate. In contrast, Petersen et al. [57] reported only 10% severe neuropathy (modified NCI-IORT criteria) avoiding overlapping fields and Krempien et al. [19] also found only an 8% neuropathy rate (RTOG criteria). In the prospective trial reported by Roeder et al. [67] no severe neuropathy (CTCAE 3.0) was found after restriction of the IORT dose to 12 Gy. Therefore overlapping fields should be avoided and the IORT dose might be restricted to 12 Gy especially if major nerves have to be included. Finally ureter stenosis has been described frequently in association with IORT in the retroperitoneal space. Miller et al. [75] thoroughly analysed this issue in an cohort of 138 patients comparing the risk for a clinically apparent ureter stenosis (defined as needing stenting, nephrostomy or surgery) irradiated ureter and the non-irradiated ureter after surgery and IORT. He observed a statistically increased 5-year incidence of 41% in the irradiated ureters compared to 19% in the non-irradiated ones. The risk was further clearly dose-dependent. Therefore one should exclude the ureters form the IORT area whenever possible or at least limit the dose although one should keep in mind that retroperitoneal surgery per se is associated with a considerable risk for ureter stenosis. However, IOERT as part of a multimodal approach offers excellent outcomes in patient with retroperitoneal STS even in prognostic unfavourable situations, especially if combined with preoperative EBRT. This approach seems more effective with regard to local control than preoperative EBRT alone and less harmful with regard to acute and late radiation related toxicities than the opposite schedule without increasing the postoperative complication rate.

## Conclusion

In summary, the combination of limb-sparing surgery, IORT and pre- or postoperative EBRT with moderate doses consistently achieved excellent local control rates in extremity STS which are at least comparable to approaches using EBRT alone but usually including patient cohorts with higher proportions of unfavourable prognostic factors. Further on, IORT containing approaches resulted in very high limb preservation rates and good functional outcome, probably related to the smaller high dose volume. In retroperitoneal STS, the combination of preoperative EBRT, surgery and IORT consistently achieved high local control rates which seem superior to surgery alone or surgery with EBRT at least with regard to local control and in some reports even to overall survival. Further on, preoperative EBRT in combination with IORT seems to be superior to the opposite combination with regard to local control and toxicity. No major differences in wound healing disturbances or postoperative complication rates can be observed with IORT compared to non-IORT containing approaches. Neuropathy of major nerves remains a dose limiting toxicity requiring dose restrictions or exclusion from target volume. Gastrointestinal structures and ureters should be excluded from the IORT area whenever possible and the IORT volume should be restricted to the available minimum. Nevertheless, IORT represents an ideal boosting method if combined with EBRT enabling the application of very high doses with low toxicities which should be further evaluated preferably in prospective randomized trials.

## Abbrevations

ADL: Activities of daily living
Ccm: Cubic centimeter
Cm: Centimeter
CTCAE: Common Toxicity Criteria for Adverse events
EBRT: External beam radiation therapy
f/u: Follow up
HDR: High dose rate
GI: Gastrointestinal
Gy: Gray
IOERT: Intraoperative electron radiation therapy
IORT: Intraoperative radiation therapy
IMRT: Intensity-modulate radiation therapy
ISIORT: International Society of Intraoperative Radiation Therapy
LC: Local control
LINAC: linear accelerator
MeV: Mega electron volts
MGH: Massachusetts General Hospital
NCI: National Cancer Institute
NCIC: National Cancer Institute Canada
NCI-IORT criteria: Toxicity criteria developed by the NCI IORT working group
OS: Overall survival
Pts: Patients
RT: Radiation therapy
RTOG: Radiation Therapy Oncology Group
STS: Soft tissue sarcoma
US: United States
WHO: World health organization

## References

[1] Jemal A, Siegel R, Ward E, Hao Y, Xu J, Thun MJ (2009). Cancer statistics 2009. CA Cancer J Clin.

[2] Lawrence W, Donegan WL, Natarajan N, Mettlin C, Beart R, Winchester D (1987). Adult soft tissue sarcomas. A pattern of care survey of the American College of Surgeons. Ann Surg.

[3] Zaho RP, Yu XL, Zhang Z, Jia LJ, Feng Y, Yang ZZ, Chen XX, Wang J, Ma SL, Guo XM (2016). The efficacy of postoperative radiotherapy in localized primary soft tissue sarcoma treated with conservative surgery. Radiat Oncol.

[4] Andrä C, Rauch J, Li M, Ganswindt U, Belka C, Saleh-Ebrahimi L, Ballhausen H, Nachbichler SB, Roeder F (2015). Excellent local control and survival after postoperative or definitive radiation therapy for sarcomas of the head and neck. Radiat Oncol.

[5] Kretzler A, Molls M, Gradinger R, Lukas P, Steinau HU, Würschmidt F (2004). Intraoperative radiotherapy of soft tissue sarcomas of the extremity. Strahlenther Onkol.

[6] Oertel S, Treiber M, Zahlten-Hinguranage A, Eichin S, Roeder F, Funk A, Hensley FW, Timke C, Niethammer AG, Huber PE, Weitz J, Eble MJ, Buchler MW, Bernd L, Debus J, Krempien RC (2006). Intraoperative electron boost radiation followed by moderate doses of external beam radiotherapy in limb-sparing treatment of patients with extremity soft-tissue sarcoma. Int J Radiat Oncol Biol Phys.

[7] Roeder FF, Goetz JM, Habl G, Bischof M, Krempien R, Buechler MW, Hensley FW, Huber PE, Weitz J, Debus J (2012). Intraoperative electron radiation therapy (IOERT) in the management of locally recurrent rectal cancer. BMC Cancer.

[8] Roeder F, Treiber M, Oertel S, Dinkel J, Timke C, Funk A, Garcia-Huttenlocher H, Bischof M, Weitz J, Harms W, Hensley FW, Buchler MW, Debus J, Krempien R (2007). Patterns of failure and local control after intraoperative electron boost radiotherapy to the presacral space in combination with total mesorectal excision in patients with locally advanced rectal cancer. Int J Radiat Oncol Biol Phys.

[9] Roeder FF, Timke C, Uhl M, Habl G, Hensley FW, Buechler MW, Krempien R, Huber PE, Debus J, Werner J (2012). Aggressive local treatment containing intraoperative radiation therapy (IORT) for patients with isolated local recurrences of pancreatic cancer: a retrospective analysis. BMC Cancer.

[10] Roeder F, Timke C, Oertel S, Hensley FW, Bischof M, Muenter MW, Weitz J, Buchler MW, Lehner B, Debus J, Krempien R (2010). Intraoperative electron radiotherapy in the management of aggressive fibromatosis. Int J Radiat Oncol Biol Phys.

[11] Roeder FF, Timke C, Saleh-Ebrahimi L, Schneider L, Hackert T, Hartwig W, Kopp-Schneider A, Hensley FW, Buechler MW, Debus J, Huber PE, Werner J (2012). Clinical phase I/II trial to investigate neoadjuvant intensity-modulated short term radiation therapy (5x5 Gy) and intraoperative radiation therapy (15 gy) in patients with primarily resectable pancreatic cancer – NEOPANC. BMC Cancer.

[12] Kawamura M, Itoh Y, Sawaki M, Kikumori T, Tsunoda N, Kamomae T, Kubota S, Okada T, Nakahara R, Ito J, Hayashi H, Naganawa S (2015). A phase I/II trial of intraoperative breast radiotherapy in an Asian population: 5-year results of local control and cosmetic outcome. Radiat Oncol.

[13] Sarmento S, Costa F, Pereira A, Lecart J, Dias A, Cunha L, Sousa O, Silva JP, Santos L (2015). Attenuation measurements show that the presence of a TacholSil surgical patch will not compromise target irradiation in intra-operative electron radiation therapy or high-dose rate brachytherapy. Radiat Oncol.

[14] Lopez-Tarjuelo J, Morillo-Macias V, Bouche-Babiloni A, Boldo-Roda E, Lozoya-Albacar R, Ferrer-Albiach C (2016). Implementation of an intraoperative electron radiotherapy in vivo dosimetry program. Radiat Oncol.

[15] Zagars GK, Ballo MT, Pisters PW, Pollock RE, Patel SR, Benjamin RS, Evans HL (2003). Prognostic factors for patients with localized soft-tissue sarcoma treated with conservation surgery and radiation therapy: an analysis of 1225 patients. Cancer.

[16] Calvo FA, Meirino RM, Orecchia R (2006). Intraoperative radiation therapy first part: rationale and techniques. Crit Rev Oncol Hematol.

[17] Kneschaurek P, Wehrmann R, Hugo C, Stepan R, Lukas P, Molls M (1995). The flab method of intraoperative radiotherapy. Strahlenther Onkol.

[18] Krempien R, Roeder F, Oertel S, Roebel M, Weitz J, Hensley FW, Timke C, Funk A, Bischof M, Zabel-Du Bois A, Niethammer AG, Eble MJ, Buchler MW, Treiber M, Debus J (2006). Long-term results of intraoperative presacral electron boost radiotherapy (IOERT) in combination with total mesorectal excision (TME) and chemoradiation in patients with locally advanced rectal cancer. Int J Radiat Oncol Biol Phys.

[19] Krempien R, Roeder F, Oertel S, Weitz J, Hensley FW, Timke C, Funk A, Lindel K, Harms W, Buchler MW, Debus J, Treiber M (2006). Intraoperative electron-beam therapy for primary and recurrent retroperitoneal soft-tissue sarcoma. Int J Radiat Oncol Biol Phys.

[20] Kirkpatrick JP, Meyer JJ, Marks LB (2008). The linearquadratic model is inappropriate to model high dose per fraction effects in radiosurgery. Semin Radiat Oncol.

[21] Park C, Papiez L, Zhang S, Story M, Timmerman RD (2008). Universal survival curve and single fraction equivalent dose: useful tools in understanding potency of ablative radiotherapy. Int J Radiat Oncol Biol Phys.

[22] Fuks Z, Kolesnick R (2005). Engaging the vascular component of the tumor response. Cancer Cell.

[23] Rosenberg SA, Tepper J, Glatstein E (1982). The treatment of soft-tissue sarcomas of the extremities. Ann Surg.

[24] Yang JC, Chang AE, Baker AR, Sindelar WF, Danforth DN, Topalian SL, DeLaney T, Glatstein E, Steinberg SM, Merino MJ, Rosenberg SA (1998). Randomized prospective study of the benefit of adjuvant radiation therapy in the treatment of soft tissue sarcomas of the extremity. J Clin Oncol.

[25] Jebsen NL, Trovik CS, Bauer HC (2008). Radiotherapy to improve local control regardless of surgical margin and malignancy grade in extremity and trunk wall soft tissue sarcoma: a Scandinavian sarcoma group study. Int J Radiat Oncol Biol Phys.

[26] O’Sullivan B, Davis AM, Turcotte R, Bell R, Catton C, Chabot P, Wunder J, Kandel R, Goddard K, Sadura A, Pater J, Zee B (2002). Preoperative versus postoperative radiotherapy in soft-tissue sarcoma of the limbs: a randomized trial. Lancet.

[27] Stinson SF, DeLaney TF, Greenberg J, Yang JC, Lampert MH, Hicks JE, Venzon D, White DE, Rosenberg SA, Glatstein EJ (1991). Acute and long-term effects on limb function of combined modality limb sparing therapy for extremity soft tissue sarcoma. Int J Radiat Oncol Biol Phys.

[28] Davis AM, O’Sullivan B, Turcotte R (2005). Late radiation morbidity following randomization to preoperative versus postoperative radiotherapy in extremity soft tissue sarcoma. Radiother Oncol.

[29] Roeder F, Lehner B, Schmitt T, Kasper B, Egerer G, Sedlaczek O, Grüllich C, Mechtersheimer G, Wuchter P, Hensley FW, Huber PE, Debus J, Bischof M (2014). Excellent local control with IOERT and postoperative EBRT in high grade extremity sarcoma: results from a subgroup analysis of a prospective trial. BMC Cancer.

[30] Roeder F, Lehner B, Saleh-Ebrahimi L, Hensley FW, Ulrich A, Alldinger I, Mechtersheimer G, Huber PE, Krempien R, Bischof M, Debus J, Uhl M (2016). Intraoperative electron radiation therapy combined with external beam radiation therapy and limb sparing surgery in extremity soft tissue sarcoma: a retrospective single center analysis of 183 cases. Radiother Oncol.

[31] Gunderson LL, Martik JK, Earle JD, Byer DE, Voos M, Fieck JM, Kvols LK, Rorie DK, Martinez A, Nagorney DM (1984). Intraoperative and external beam irradiation with or without resection: Mayo pilot experience. Mayo Clin Proc.

[32] Petersen IA, Krempien R, Beauchamp C, Eble M, Calvo FA, Azinovic I, Callister MD, Alvarez A. Extremity and trunk soft-tissue sarcomas. In Gunderson LL, Willett CG, Calvo FA, Harrisson LB (editors): Intraoperative Irradiation, second edition. New York: Humana Press; 2011. p. 397.

[33] Edmonson JH, Petersen IA, Shives TC, Mahoney MR, Rock MG, Haddock MG, Sim FH, Maples WJ, O’Connor MI, Gunderson LL, Foo ML, Pritchard DJ, Buckner JC, Stafford SL (2001). Chemotherapy, irradiation, and surgery for function-preserving therapy of primary extremity soft-tissue sarcomas. Cancer.

[34] Azinovic I, Martinez Monge R, Javier Aristu J, Salgado E, Villafranca E, Hidalgo OF, Amillo S, San Julian M, Villas C, Aramendia JM, Calvo FA (2003). Intraoperative radiotherapy electron boost followed by moderate doses of external beam radiotherapy in resected soft-tissue sarcomas of the extremities. Radiother Oncol.

[35] Llacer C, Delannes M, Minsat M, Stoeckle E, Votron L, Martel P, Bonnevialle P, Bui BG, Chevreau C, Kantor G, Daly-Schveitzer N, Thomas L (2006). Low-dose intraoperative brachytherapy in soft tissue sarcomas involving neurovascular structure. Radiother Oncol.

[36] Alvarez A, Calvo FA, Gonzales C, Ferrer M, Lozano MA, Calin A, Casteleiro R, Alvarez E, Pedrero F, Herranz R (2008). IORT in soft tissue sarcomas involving extremities: toxicities and long-term functional results [abstract]. Revisiones en cancer.

[37] Callister MD, Beauchamp CP, Fitch TR, Gunderson LL (2008). Preoperative radiation and IOERT for soft-tissue sarcomas of the extremities and trunk [abstract]. Revisiones en cancer.

[38] Schmitt T, Lehner B, Kasper B, Bischof M, Roeder F, Dietrich S, Dimitrakopoulou-Strauss A, Strauss LG, Mechtersheimer G, Wuchter P, Ho AD, Egerer G (2011). A phase II study evaluating neo-/adjuvant EIA chemotherapy, surgical resection and radiotherapy in high-risk soft tissue sarcomas. BMC Cancer.

[39] Roeder F, De Paoli A, Alldinger I, Bertola G, Boz G, Garcia-Sabredo JL, Uhl M, Alvarez A, Lehner B, Calvo FA, Krempien R (2015). IORT after gross total resection combined with EBRT in extremity soft tissue sarcoma: a pooled analysis [abstract]. Radiother Oncol.

[40] Calvo FA, Sole CV, Polo A, Cambeiro M, Montero A, Alvarez A, Cuervo M, San Julian M, Martinez-Monge R (2014). Limb-sparing management with surgical resection, external-beam and intraoperative electron-beam radiation therapy for boost for patients with primary soft tissue sarcoma of the extremity: a multicentric pooled analysis of long-term outcomes. Strahlenther Onkol.

[41] Krempien R, Roeder F, Buchler MW, Di Paoli A, Bertola G, Boz G, Ferrer M, Alvarez A, Calvo FA (2008). Intraoperative Radiation Therapy (IORT) For Primary and recurrent extremity soft tissue sarcoma : First results of a pooled analysis [abstract]. Revisiones en Cancer.

[42] Hui AC, Ngan SY, Wong K, Powell G, Choong PF (2006). Preoperative radiotherapy for soft tissue sarcoma: the Peter MacCallum Cancer Centre Experience. Eur J Surg Oncol.

[43] Dagan R, Indelicato DJ, McGee L, Morris CG, Kirwan JM, Knapik J, Reith J, Scarborough MT, Gibbs CP, Marcus RB, Zlotecki RA (2012). The significance of a marginal excision after preoperative radiation therapy of soft tissue sarcoma of the extremity. Cancer.

[44] MacDermed DM, Miller LL, Peabody TD, Simon MA, Luu HH, Haydon RC, Montag AG, Undevia SD, Connell PP (2010). Primary tumor necrosis predicts distant control in locally advanced soft tissue sarcomas after preoperative concurrent chemoradiotherapy. Int J Radiat Oncol Biol Phys.

[45] Kraybill WG, Harris J, Spiro IJ, Ettinger DS, DeLaney TF, Blum RH, Lucas DR, Harmon DC, Letson GD, Eisenberg B (2006). Phase II Study of neoadjuvant chemotherapy and radiation therapy in the management of high-risk, high-grade, soft tissue sarcomas of the extremities and body wall: radiation therapy oncology group trial 9514. J Clin Oncol.

[46] Felderhof JM, Creutzberg CL, Putter H, Nout RA, Bovee JV, Dijkstra PD, Hartgrink HH, Marijnen CA (2012). Long term clinical outcome of patients with soft-tissue sarcomas treated with limb-sparing surgery and postoperative radiotherapy. Acta Oncol.

[47] Alektiar KM, Brennan MF, Singer S (2011). Local Control comparison of adjuvant brachytherapy to intensity-modulated radiotherapy in primary high-grade sarcoma of the extremity. Cancer.

[48] Lee J, Park YJ, Yang DS, Yoon WS, Lee JA, Rim CH, Kim CY (2012). Treatment outcome of conservative surgery plus postoperative radiotherapy for extremity soft tissue sarcoma. Radiat Oncol J.

[49] Sampath S, Schultheiss TE, Hitchcock YJ, Randall RL, Shrieve DC, Wong JY (2011). Preoperative versus postoperative radiotherapy in soft-tissue sarcoma: multi-institutional analysis of 821 patients. Int J Radiat Oncol Biol Phys.

[50] Folkert M, Singer S, Brennan MF, Kuk D, Qin LX, Kobayashi WK, Crago AM, Alektiar KM (2014). Comparison of local recurrence with conventional and intensity-modulated radiation therapy for primary soft-tissue sarcomas of the extremity. J Clin Oncol.

[51] Kunos C, Colussi V, Getty P, Kinsella T (2006). Intraoperative Electron Radiotherapy for Extremity Sarcomas does not increase acute or late morbidity. Clin Orthop Rel Res.

[52] Sindelar WF, Kinsella TJ, Chen PW, DeLaney TF, Tepper JE, Rosenberg SA, Glatstein E (1993). Intraoperative radiotherapy in retroperitoneal sarcomas. Final results of a prospective, randomized clinical trial. Arch Surg.

[53] Gunderson LL, Nelson H, Martenson JA, Cha S, Haddock M, Devine R, Fieck JM, Wolff B, Dozois R, O’Connell MJ (1997). Locally advanced primary colorectal cancer: intraoperative electron and external beam irradiation +/− 5-FU. Int J Radiat Oncol Biol Phys.

[54] Haddock MG, Miller RC, Nelson H, Pemberton JH, Dozois EJ, Alberts SR, Gunderson LL (2011). Combined modality therapy including intraoperative electron irradiation for locally recurrent colorectal cancer. Int J Radiat Oncol Biol Phys.

[55] van Kampen M, Eble MJ, Lehnert T, Bernd L, Jensen K, Hensley F, Krempien R, Wannenmacher M (2001). Correlation of intraoperatively irradiated volume and fibrosis in patients with soft-tissue sarcoma of the extremities. Int J Radiat Oncol Biol Phys.

[56] Roeder FF, Schulz-Ertner D, Nikoghosyan AV, Huber PE, Edler L, Habl G, Krempien R, Oertel S, Saleh-Ebrahimi L, Hensley FW, Buechler MW, Debus J, Koch M, Weitz J (2012). Bischof M.A clinical phase I/II trial to investigate preoperative dose-escalated intensity-modulated radiation therapy (IMRT) and intraoperative radiation therapy (IORT) in patients with retroperitoneal soft tissue sarcoma. BMC Cancer.

[57] Petersen IA, Haddock MG, Donohue JH, Nagorney DM, Grill J, Sargent DJ, Gunderson LL (2002). Use of intraoperative electron beam radiotherapy in the management of retroperitoneal soft tissue sarcomas. Int J Radiat Oncol Biol Phys.

[58] Pawlik TM, Ahuja N, Herman JM (2007). The role of radiation in retroperitoneal sarcomas: a surgical perspective. Curr Opin Oncol.

[59] Catton CN, O’Sullivan B, Kotwall C, Cummings B, Hao Y, Fornasier V (1994). Outcome and prognosis in retroperitoneal soft tissue sarcoma. Int J Radiat Oncol Biol Phys.

[60] Heslin MJ, Lewis JJ, Nadler E, Newman E, Woodruff JM, Casper ES, Leung D, Brennan MF (1997). Prognostic factors associated with long-term survival for retroperitoneal sarcoma: implications for management. J Clin Oncol.

[61] Lewis JJ, Leung D, Woodruff JM, Brennan MF (1998). Retroperitoneal soft-tissue sarcoma: Analysis of 500 patients treated and followed at a single institution. Ann Surg.

[62] Stoeckle E, Coindre JM, Bonvalot S, Kantor G, Terrier P, Bonichon F, Nguyen Bui B, French Federation of Cancer Centers Sarcoma Group (2001). Prognostic factors in retroperitoneal sarcoma: A multivariate analysis of a series of 165 patients of the French Cancer Center Federation Sarcoma Group. Cancer.

[63] Tepper JE, Suit HD, Wood WC, Proppe KH, Harmon D, McNulty P (1984). Radiation therapy of retroperitoneal soft tissue sarcomas. Int J Radiat Oncol Biol Phys.

[64] Fein DA, Corn BW, Lanciano RM, Herbert SH, Hoffman JP, Coia LR (1995). Management of retroperitoneal sarcomas: Does dose escalation impact on locoregional control ?. Int J Radiat Oncol Biol Phys.

[65] Dziewirski W, Rutkowski P, Nowecki ZI, Salamacha M, Morysinski T, Kulik A, Kawczynska M, Kasprowicz A, Lyczek J, Ruja W (2006). Surgery combined with intraoperative brachytherapy in the treatment of retroperitoneal sarcomas. Ann Surg Oncol.

[66] Alektiar KM, Hu K, Anderson L, Brennan MF, Harrison LB (2000). High-dose-rate intraoperative radiation therapy (HDR-IORT) for retroperitoneal sarcomas. Int J Radiat Oncol Biol Phys.

[67] Roeder F, Ulrich A, Habl G, Uhl M, Saleh-Ebrahimi L, Huber PE, Schulz-Ertner D, Nikoghosyan AV, Alldinger I, Krempien R, Mechtersheimer G, Hensley FW, Debus J, Bischof M (2014). Clinical phase I/II trial to investigate preoperative dose-escalated intensity-modulated radiation therapy (IMRT) and intraoperative radiation therapy (IORT) in patients with retroperitoneal soft tissue sarcoma: interim analysis. BMC Cancer.

[68] Gieschen HL, Spiro IJ, Suit HD, Ott MJ, Rattner DW, Ancukiewicz M, Willett CG (2001). Long-term results of intraoperative electron beam radiotherapy for primary and recurrent retroperitoneal soft tissue sarcoma. Int J Radiat Oncol Biol Phys.

[69] Pierie JP, Betensky RA, Choudry U, Willett CG, Souba WW, Ott MJ (2006). Outcomes in a series of 103 retroperitoneal sarcomas. EJSO.

[70] Pawlik TM, Pisters PW, Mikula L, Feig B, Hunt KK, Cormiert JN, Ballo MT, Catton CN, Jones JJ, O’Sullivan B, Pollock RE, Swallow CJ (2006). Long-term results of two prospective trials of preoperative external beam radiotherapy for localized intermediate- or high-grade retroperitoneal soft tissue sarcoma. Ann Surg Oncol.

[71] Ballo MT, Zagars GK, Pollock RE, Benjamin RS, Feig BY, Cormier JN, Hunt KK, Patel SR, Trent JC, Beddar S, Pisters PW (2007). Retroperitoneal soft tissue sarcoma: an analysis of radiation and surgical treatment. Int J Radiat Oncol Biol Phys.

[72] Sweeting RS, Deal AM, Llaguna OH, Bednarski BK, Meyers MO, Yeh JJ, Calvo BF, Tepper JE, Kim HJ (2013). Intraoperative electron radiation therapy as an important treatment modality in retroperitoneal sarcoma. J Surg Res.

[73] Gronchi A, De Paoli A, Dani C, Merlo DF, Quagliuolo V, Grignani G, Bertola G, Navarria P, Sangalli C, Buonadonna A, De Sanctis R, Sanfilippo R, Dei Tos AP, Stacchiotti S, Giorello L, Fiore M, Bruzzi P, Casali PG (2014). Preoperative chemo-radiation therapy for localised retroperitoneal sarcoma: a phase I-II study from the Italian Sarcoma Group. Eur J Cancer.

[74] Van de Voorde L, Delrue L, van Eijkeren M, de Meerleer G (2011). Radiotherapy and surgery – an indispensable duo in the treatment of retroperitoneal sarcoma. Cancer.

[75] Miller RC, Haddock MG, Petersen IA, Gundersson LL, Furth AF (2006). Intraoperative electron-beam radiotherapy and ureteral obstruction. Int J Radiat Oncol Biol Phys.

[76] Bobin JY, Al-Lawati T, Granero LE, Adham M, Romestaing P, Chapet O, Issac S, Gerard JP (2003). Surgical management of retroperitoneal sarcomas associated with external and intraoperative electron beam radiotherapy. Eur J Surg Oncol.

